# CD20+ and CD204+ exhibit distinct prognostic associations in thymic epithelial tumors

**DOI:** 10.3389/fonc.2025.1710544

**Published:** 2025-11-18

**Authors:** Qinyang Chen, Jian Zhang, Jianwei Feng, Chaowen Chen, Chuan Su, Wenxiu Yao

**Affiliations:** 1 The Academy of Chinese Health Risks, West China Hospital, Sichuan University, Chengdu, China; 2 Department of Oncology, The Third People's Hospital of Yibin, Yibin, China; 3 Department of Oncology, University of Electronic Science and Technology of China, Sichuan Cancer Hospital and Institute & Cancer, The Second People's Hospital of Sichuan Province, Chengdu, China

**Keywords:** thymic epithelial tumors, tumor microenvironment, tumor-associated macrophages, CD20, CD204

## Abstract

**Introduction:**

Thymic epithelial tumors (TETs), encompassing thymomas and thymic carcinomas, are rare malignancies originating from thymic epithelial cells. This study aimed to characterize tumor-infiltrating immune cells (TIIC) within the tumor microenvironment (TME) and evaluate their prognostic significance in TETs.

**Methods:**

We retrospectively analyzed 125 patients with surgically resected TETs (2009-2021). Immunohistochemical staining of tumor specimens was performed to assess TIIC distribution, with significant associations between immune markers and survival outcomes evaluated using Cox regression.

**Results:**

Among the analyzed markers, CD20, CD204, CD206, and CD47 emerged as potential predictors for disease-free survival (DFS), while CD20 and CD204 showed prognostic relevance for overall survival (OS). Specifically, stromal CD20+ TIIC density was independently associated with prolonged DFS(HR=4.74, 95% CI(1.673-13.434), P=0.003) and OS (HR=5.086, 95% CI(1.391-18.594), P=0.014), whereas elevated CD204+ TIIC infiltration correlated with reduced DFS(HR=0.154, 95% CI(0.043-0.547), P=0.004) and OS(HR=0.169, 95% CI(0.056-0.607), P=0.002).

**Conclusion:**

These findings suggest that targeting M2 macrophage-driven immunosuppression may enhance therapeutic efficacy in TETs.

## Introduction

Thymic epithelial tumors (TETs) are a group of rare neoplasms that originate from the epithelial cells of the thymus gland. This category primarily encompasses thymomas, which exhibit relatively indolent biological behavior, and the more aggressive thymic carcinomas. Although thymic epithelial tumors (TETs) are relatively rare, they are the most common tumors in the anterior mediastinum, with thymic carcinoma having a five-year survival rate of only 46.2% (1). For advanced TETs, radiochemotherapy is often employed to prolong disease control (2). However, TETs typically exhibit indolent clinical behavior and often respond poorly to chemotherapy, making it difficult to achieve optimal therapeutic outcomes (3). Additionally, the low incidence rate of TETs, coupled with their diverse and complex histological types and the lack of uniformity between disease progression and clinical prognosis, results in a relatively limited clinical understanding of TETs. Therefore, the diagnosis, treatment, and prognostic assessment of TETs remain a clinical challenge.

The thymus is a critical immune organ in the human body, playing a pivotal role in immune responses and regulation (4). The relative balance of immune effector cells and suppressor populations within the tumor microenvironment (TME) may contribute to determining the fate of tumors (5). Consequently, strategies targeting the TME may emerge as a novel and essential approach to enhancing the efficacy of cancer therapies (6). In the TME, the primary mediators of anti-tumor immune responses are lymphocytes and tumor-associated macrophages (TAMs). Research evidence indicates that adaptive immunity, mediated by T and B lymphocytes, constitutes an effective and sustained defense mechanism against tumors (7). Among T cell populations, cytotoxic CD8+ T cells can reside in and eliminate tumors (8). These cells exert their anti-tumor effects by secreting cytotoxic molecules and cytokines, including perforin, granzymes, interferon-γ (IFN-γ), and tumor necrosis factor-α (TNF-α) (9).

CD20 is a specific marker widely expressed on the surface of B lymphocytes at various stages of differentiation, playing an essential role in B cell development, differentiation, and activation, and is also a key therapeutic target for treating B cell malignancies (10). An increasing number of studies suggest that B cells possess anti-tumor activity, highlighting their potential in cancer immunotherapy. The B-cell populations detected in the thymic tissue of this study may represent immune cells residing within or infiltrating the tumor microenvironment.

Tumor-associated macrophages (TAMs) can be categorized into two primary phenotypes based on their activation status: the classically activated M1 macrophages and the alternatively activated M2 macrophages. M1 macrophages are characterized by their ability to kill tumor cells, promote inflammation, and inhibit tumor growth, whereas M2 macrophages are involved in tissue remodeling, suppression of adaptive immunity, and the promotion of angiogenesis and tumor growth (11). Additionally, TAMs can directly or indirectly suppress T cell responses in the tumor microenvironment (TME) through various mechanisms. They produce inhibitory cytokines such as interleukin-10 (IL-10) and transforming growth factor-beta (TGF-β), participate in immune checkpoints through the expression of molecules like programmed death-ligand 1 (PD-L1), and affect T cell metabolic activities, including the consumption of metabolites and the production of reactive oxygen species. Indirectly, they modulate cellular responses within the immune microenvironment, including the recruitment of immunosuppressive cells (such as regulatory T cells, Tregs) or the inhibition of stimulatory cells (like dendritic cells) (12). TAMs play a significant role in cancer initiation, progression, and metastasis and are closely associated with poor prognosis in various types of cancer (13). Targeting TAMs may offer considerable benefits for cancer therapy.

CD204 and CD206 are specific markers on M2 macrophages. CD204, also known as scavenger receptor A, is an immune receptor highly expressed on M2 macrophages, forming homotrimers on the cell surface and participating in various biological pathways, making it a biomarker with negative prognostic value. CD206, also known as the macrophage mannose receptor, is a C-type lectin commonly expressed by macrophages and dendritic cells in tissues (14). Additionally, the membrane surface of M2 macrophages can express a signaling regulatory protein α (SIRPα), which binds to its ligand CD47 and plays a role in dampening innate immune responses (15). In the tumor immune microenvironment, cancer cells often evade immune system clearance and suppress the immunogenic processing of cancer antigens by overexpressing CD47, which interferes with the phagocytic activity of macrophages (16). Targeted blockade of the interaction between CD47 and SIRPα can potentially enhance the phagocytic activity of macrophages against tumor cells, thereby promoting the body’s immune response to the tumor. An increasing number of CD47 inhibitors are being used in cancer treatment and have entered clinical trials (17–19).

Currently, there is a lack of clear clinical guidelines for the treatment of thymic epithelial tumors (TETs). In the era of precision medicine, although clinical trial results for immunotherapies targeting TETs have been less than optimal and are associated with high rates of adverse reactions, the search for appropriate immunotherapy approaches remains crucial. The tumor microenvironment (TME) is widely recognized as a critical battleground for anti-tumor immune responses. Exploring the diagnosis, treatment, and prognosis of thymic tumors from the perspective of the TME may yield breakthrough discoveries. Assessing the tumor immune landscape can reveal the prognostic and predictive value of immune-related biomarkers, enhancing our understanding of tumor behavior. This study sets the stage for future research on more effective cancer treatment, providing new insights into disease-related characteristics. Its results offer a useful reference for cancer therapeutic approaches in clinical practice.

### Study population

A retrospective analysis was conducted on 170 patients with thymic epithelial tumors (TETs) who were diagnosed and underwent surgical treatment at a tertiary hospital in Sichuan, China, between 2009 and 2021, with complete clinical records. The final cohort for follow-up analysis consisted of approximately 125 cases.

Inclusion Criteria:

1. Patients had not received any preoperative anti-tumor treatments such as radiotherapy, chemotherapy, targeted therapy, or immunotherapy.
2. Age range: 18 to 70 years old.
3. Pathological confirmation of thymic origin with sufficient sample availability for analysis.
4. Eastern Cooperative Oncology Group (ECOG) performance status score of 0 to 2.
5. Patients had not been treated with thymosin alpha preoperatively.
6. No history of other tumors aside from TETs.
7. Patients underwent regular post-treatment check-ups and were available for follow-up.

Exclusion Criteria:

1. Patients with a history of severe organic or functional organ diseases.
2. Pregnant or breastfeeding women.
3. Patients who could not undergo regular postoperative check-ups or had incomplete follow-up data.
4. Patients with severe mental disorders.
5. Patients under 18 years of age or over 70 years of age.
6. Patients deemed unsuitable for inclusion by the investigators for other reasons.

This rigorous selection process ensured that the study cohort was well-defined and suitable for the analysis of clinical outcomes and the evaluation of treatment efficacy in patients with TETs.

## Methods

**Figure 1:**
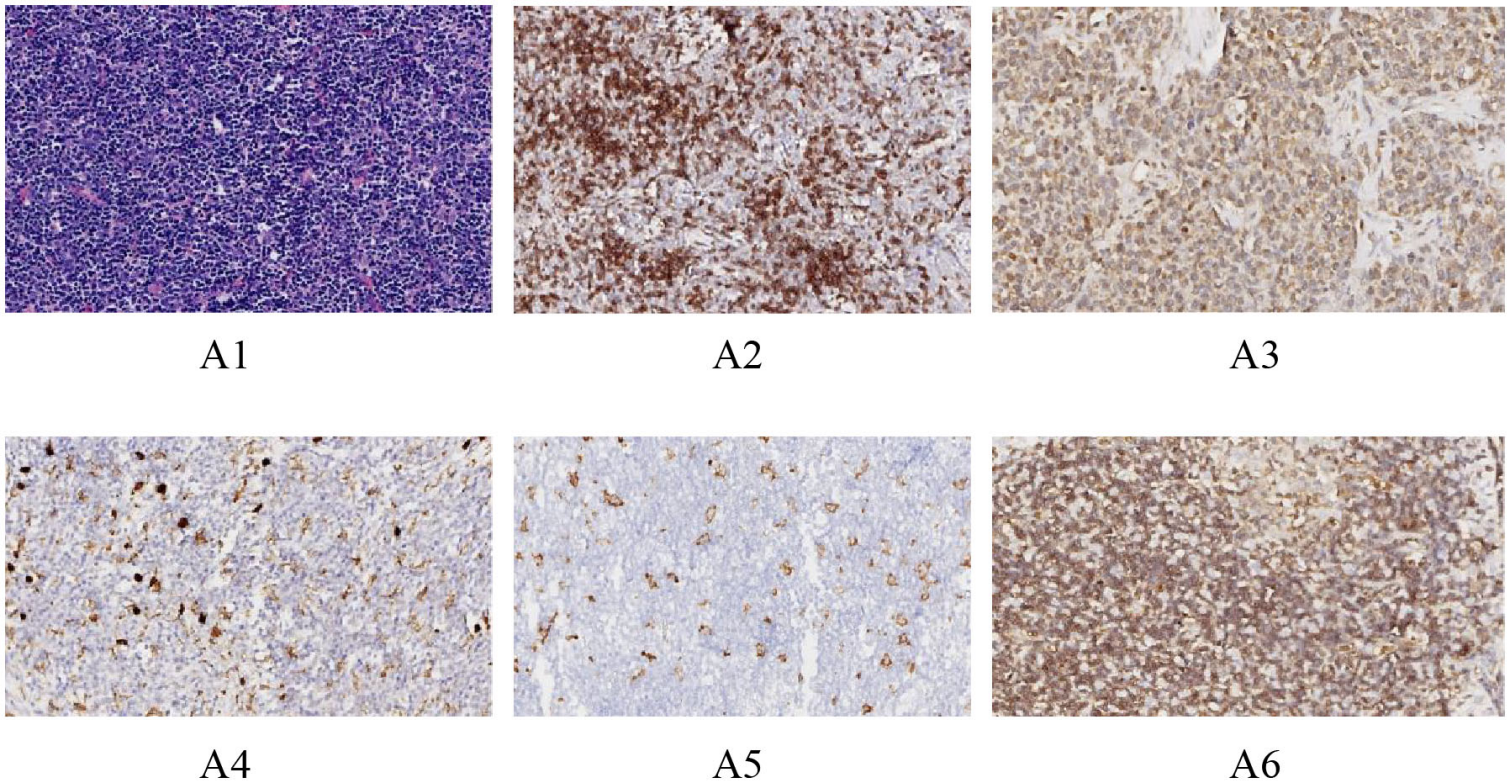
Immunohistochemical images of different antibodies. (A1: HE staining results (20X). A2: Positive results of CD8 staining (20X). A3: Positive CD20 staining results (20X). A4: Positive CD204 staining results (20X).A5: Positive CD206 staining results (20X).A6: Positive CD47 staining results (20X).).

Paraffin-embedded thymic tumor blocks were subjected to routine sectioning and hematoxylin-eosin (H&E) staining. Preliminary tests were conducted to determine the optimal dilution concentrations for the primary antibodies against CD8(Gene Tex,GTX74773),CD20(MXB,2112170020h),CD204,(Bio-techne,AF2708),CD206(Bio-techne,MAB25341),andCD47(Bio-techne,AF4670), prior to performing immunohistochemical (IHC) staining. The optimal dilutions were 1:100, 1:400, 1:150, 1:200,and 1:150, respectively. The results were interpreted under an optical microscope. Positive control slides were provided by the reagent company. Six random high-power fields (20X) were selected, and the number of positive cells was counted in each field to calculate the percentage of positive cells, with the average value taken (**Figure 1** see below). The IHC staining results were jointly assessed by two experienced pathologists under the principle of double-blind evaluation. In cases of discrepancy, a consensus was reached through discussion to determine the final percentage.

This study has two primary endpoints: overall survival (OS) and disease-free survival (DFS). OS refers to the time from randomization until death from any cause. OS is the only endpoint based solely on survival events. DFS is defined as the time from randomization to disease recurrence or death from any cause, primarily assessing disease relapse. Following this principle, all patients were followed up until 30/06/2022, with follow-up durations ranging from 1 to 11 years, and an average follow-up time of 6 years. At the end of the follow-up period in this study, all 125 cases completed the endpoint evaluation, with a loss to follow-up rate of 0%. There was no selective loss to follow-up bias, no missing data issues, and no special data handling methods were applied.

A retrospective investigation and analysis method was employed to obtain demographic data and clinical information before, during, and after treatment from hospital medical records and case management files. The collected data included age, sex, ECOG performance status, presence of myasthenia gravis, histological classification of the case, Masaoka staging, postoperative chemotherapy, postoperative radiotherapy, disease-free survival (DFS), and overall survival (OS). A total of 170 patients were included in the study, and clinical data and survival status were obtained for 125 patients through telephone follow-ups. Among the 125 cases of thymic epithelial tumors, there were 57 males and 68 females, with ages ranging from 18 to 70 years and an average age of 44 years. Sixteen cases were accompanied by myasthenia gravis, while 109 were not. The follow-up period ranged from 1 to 11 years, with an average follow-up time of 6 years. Twenty patients experienced recurrence or metastasis, 12 patients died due to the disease, and 1 patient died due to an accident. All 125 thymic epithelial tumor cases were classified according to the 2015 World Health Organization Classification of thymic epithelial tumors (4th edition) by two senior physicians who reviewed the slides separately and then discussed the classification together. Staging was performed using the Masaoka-Koga staging system (detailed version by ITMIG, the International Thymic Malignancy Interest Group).Among all the cases, there were 9 cases of type A thymoma, 36 cases of type AB thymoma, 12 cases of type B1 thymoma, 35 cases of type B2 thymoma, 13 cases of type B3 thymoma, and 20 cases of thymic carcinoma.

Statistical analysis was performed using SPSS version 23.0. Quantitative data were expressed as medians and ranges, while categorical data were presented as frequencies and proportions. The significance level for testing was set at α = 0.05. The area under the receiver operating characteristic (ROC) curve was used to calculate the Youden index for biomarkers CD8, CD20, CD204, CD206, and CD47. The relationship between these biomarkers and disease-free survival (DFS) and overall survival (OS) was assessed using Spearman’s correlation analysis, with scatter plots drawn to visually judge the correlation. Kaplan-Meier (K-M) curves were used to plot the cumulative survival function and perform survival analysis, with the log-rank test applied to determine differences in survival. Univariate analysis was conducted to identify prognostic factors for thymic epithelial tumors. Variables that showed statistical significance in the univariate analysis were included in the multivariate regression analysis model. A COX proportional hazards regression model was used for multivariate analysis to determine independent prognostic risk factors. The Kruskal-Wallis H test was employed to assess differences in the expression of CD8, CD20, CD204, CD206, and CD47 across different histological types and stages. *Post-hoc* pairwise comparisons between groups were adjusted for significance using the Bonferroni method. A P-value of less than 0.05 was considered to indicate statistical significance (**Table 1** see below).

**Table 1:** Baseline characteristics of the study participants with thymic epithelial tumors.

Variables	CD20			CD204			CD206			CD47		
	Positive (N = 116)	Negative (N = 9)	*P*	Positive (N = 50)	Negative (N = 70)	*P*	Positive (N = 18)	Negative (N = 107)	*P*	Positive (N = 92)	Negative (N = 33)	*P*
Sex												
*Female*	63 (54.31)	5 (55.56)	0.942	28 (56.00)	40 (57.14)	0.487	7 (38.89)	61 (57.01)	0.153	45 (48.91)	23 (69.70)	**0.040**
*Male*	53 (45.69)	4 (44.44)		27 (54.00)	30 (42.86)		11 (61.11)	46 (42.99)		47 (51.09)	10 (30.30)	
Myasthenia gravis (Yes)	16 (13.79)	0 (0)	0.233	7 (14.00)	9 (12.86)	0.856	1 (5.56)	15 (14.02)	0.375	12 (13.04)	4 (12.12)	0.892
Postoperative radiotherapy (Yes)	38 (32.76)	7 (77.78)	**0.007**	29 (58.00)	16 (22.86)	**<0.001**	12 (66.67)	33 (30.84)	**0.003**	41 (44.57)	4 (12.12)	**0.001**
Postoperative chemotherapy (Yes)	26 (22.41)	5 (55.56)	**0.027**	24 (48.00)	7 (10.00)	**<0.001**	11 (61.11)	20 (18.69)	**<0.001**	27 (29.35)	4 (12.12)	**0.049**
Masaoka stage												
*I*	62 (53.45)	1(11.11)	**0.014**	10 (20.00)	53 (75.71)	**0.000**	1 (5.56)	62 (57.94)	**<0.001**	–	–	–
*II*	12 (10.34)	2 (22.22)	0.276	9 (18.00)	5 (7.14)	0.068	2 (11.11)	12 (11.21)	0.990	–	–	
*III*	22 (18.97)	4 (44.44)	0.070	21 (42.00)	5 (7.14)	**<0.001**	10 (55.56)	16 (14.95)	**<0.001**	–	–	–
*IV*	20 (17.24)	2 (22.22)	0.705	15 (30.00)	7 (10.00)	**0.005**	5 (27.78)	17 (15.89)	0.220	–	–	–
Histological type (4th edition)												
*a+ab*	44 (37.93)	1 (11.11)	0.106	7 (14.00)	38 (54.29)	**<0.001**	0 (0)	45 (42.06)	**<0.001**	–	–	–
*b1*	12 (10.34)	0 (0)	0.310	2 (4.00)	10 (14.29)	0.064	0 (0)	12 (11.21)	0.135	–	–	–
*b2*	34 (29.31)	1 (11.11)	0.241	15 (30.00)	20 (28.57)	0.865	1 (5.56)	34 (31.78)	0.013	–	–	–
*b3*	11 (9.48)	2 (22.22)	0.228	11 (22.00)	2 (2.86)	**<0.001**	4 (22.22)	9 (8.41)	0.076	–	–	–
*Cancer*	15 (12.93)	5 (55.56)	**<0.001**	20 (40.00)	0 (0)	**<0.001**	13 (72.22)	7 (6.54)	**0.000**	–	–	–

The values are reported as number (%) for categorical variables at the time of enrollment into the study.The bold values indicate that the p-values are statistically significant.

## Results

Spearman correlation analysis (**Table 2**) revealed significant associations between the expression of CD20 and CD204 and both disease-free survival (DFS) and overall survival (OS). The expression of CD206 and CD47 was significantly correlated with DFS but not with OS, while CD8 expression showed no significant correlation with either DFS or OS. The log-rank test (**Table 3**) confirmed the results of the Spearman correlation analysis. For CD20, the cut-off indices for OS and DFS were 0.453 and 0.488, respectively, with significant correlations indicated by P values of 0.006 for OS and less than 0.001 for DFS. Lower percentages of CD20 expression were associated with poorer OS and DFS in patients. For CD204, the cut-off indices for OS and DFS were 0.462 and 0.488, respectively, with significant correlations shown by P values of 0.023 for OS and less than 0.001 for DFS. Higher percentages of CD204 expression were linked to poorer OS and DFS. CD206 expression had a cut-off index of 0.581 for DFS, with a highly significant correlation (P<0.001), indicating that higher percentages of CD206 expression were associated with poorer DFS. Similarly, CD47 expression had a cut-off index of 0.388 for DFS, and results showed a significant correlation (P = 0.002), suggesting that higher percentages of CD47 expression were related to poorer DFS in patients.

**Table 2:** Spearman correlation analysis to evaluate the relationship between related biomarkers and DFS and OS.

Biomarker	*P*(DFS)	*P*(OS)
CD8	0.593	0.975
CD20	0.004	0.021
CD204	0.018	0.042
CD206	0.026	0.240
CD47	0.023	0.102

**Table 3:** Univariate analysis of prognostic factors in patients with thymic epithelial tumor.

Variables	*P*(DFS)	*P*(OS)
Sex	0.246	0.473
Myasthenia gravis (Yes)	0.089	0.188
Postoperative radiotherapy	0.02	0.239
Postoperative chemotherapy (Yes)	0.519	0.596
Masaoka-Koga stage	0.041	0.022
Histological type (4th edition)	0.002	0.273
CD20	0.006	<0.001
CD204	<0.001	0.023
CD206	<0.001	–
CD47	0.002	–

Furthermore, prognostic relevance analysis of clinical factors in patients (**Table 3**) using the log-rank test found that gender, presence of myasthenia gravis, and postoperative chemotherapy were not significantly associated with DFS or OS. However, postoperative radiotherapy (P = 0.02), Masaoka staging (P = 0.002), and histological classification (P = 0.041) showed statistically significant differences in relation to DFS but not OS (**Supplementary Figure**).

**Figure 2:**
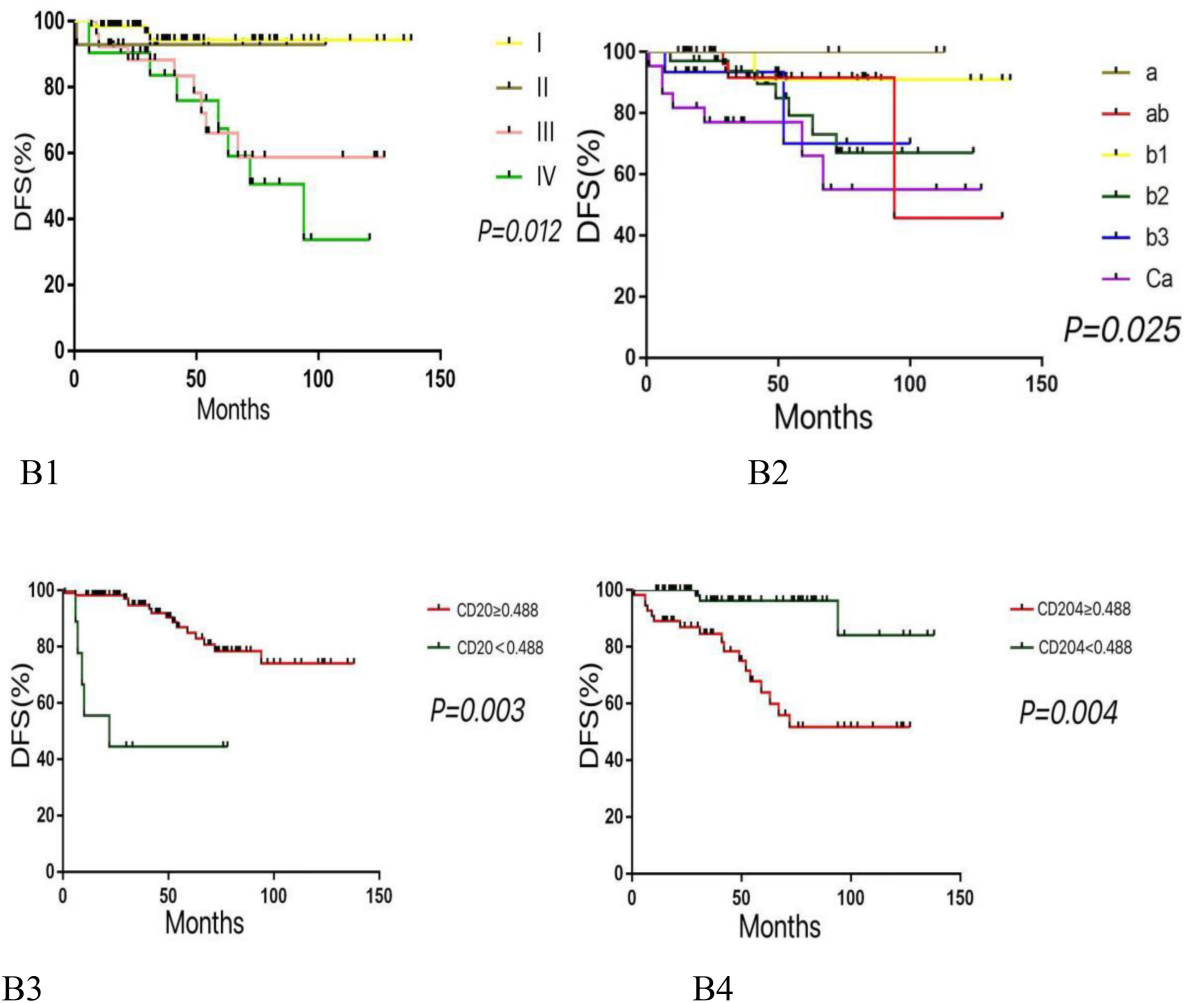
Kaplan-meier survival curves for DFS according to various variables. (B1: Higher histological subtypes are associated with lower DFS. B2: Advanced tumor stages correlate with reduced DFS. B3: Lower expression levels of CD20 are linked to decreased DFS. B4: Higher expression levels of CD204 are associated with lower DFS.).

**Figure 3:**
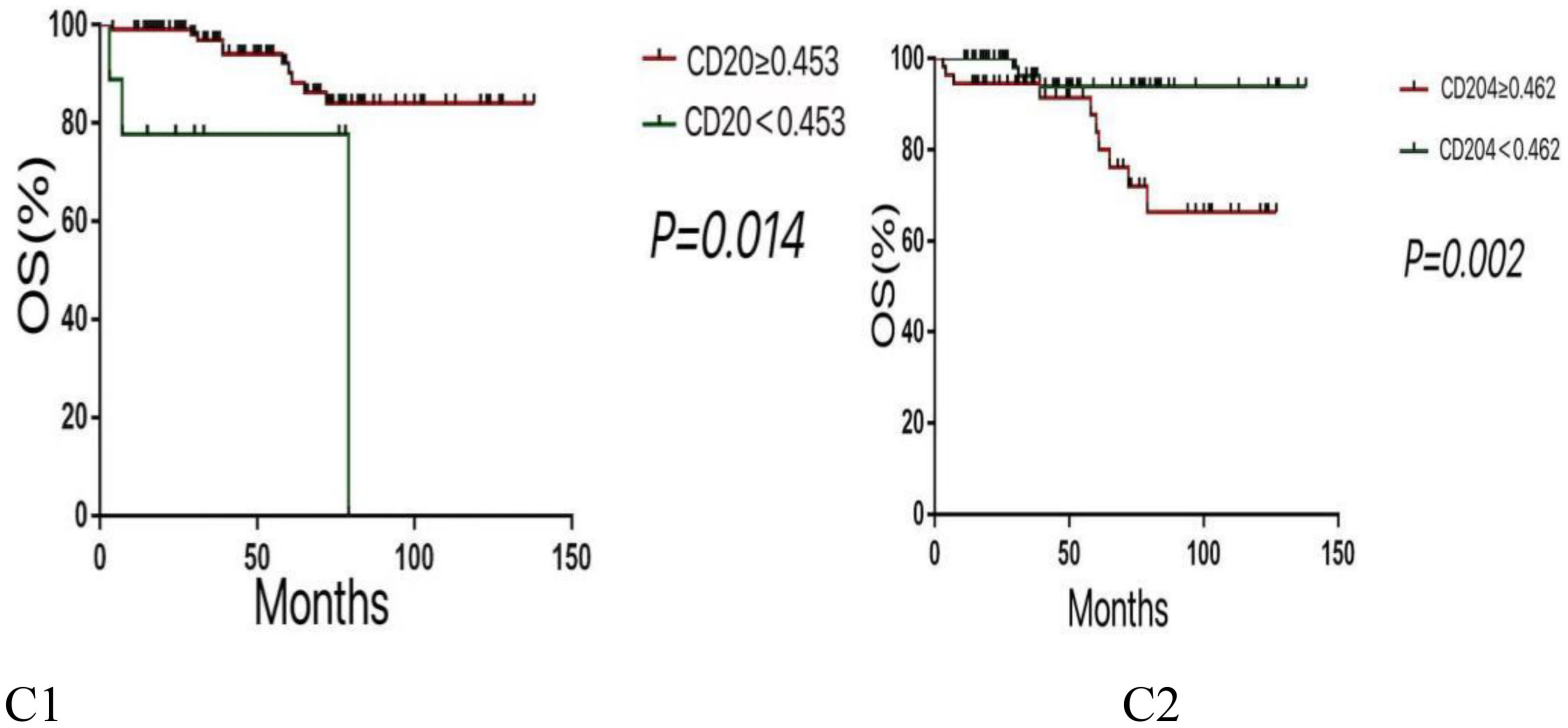
Kaplan-Meier survival curves for OS according to various variables. (C1:Lower expression levels of CD20 are associated with poorer OS. C2:Higher expression levels of CD204 are linked to reduced OS.).

Multivariate analysis using the COX proportional hazards regression model (**Table 4**) identified stage as an independent prognostic factor for disease-free survival (DFS) in thymic epithelial tumors (TETs), with P = 0.012 and a hazard ratio (HR) of 1.777. Histological type was also an independent prognostic factor for DFS in TETs, with P = 0.025 and an HR of 1.758. The expression of CD20 was found to be an independent prognostic factor for both DFS and overall survival (OS) in TETs, with P values of 0.003 (DFS) and 0.014 (OS), and hazard ratios of 4.74 (DFS) and 5.086 (OS), respectively. Similarly, the expression of CD204 was identified as an independent prognostic factor for DFS and OS in TETs, with P values of 0.004 (DFS) and 0.002 (OS), and hazard ratios of 0.154 (DFS) and 0.169 (OS), respectively. COX regression survival curves are presented in **Figures 2**, **3**.

**Table 4:** Multivariate analysis of prognostic factors in patients with thymic epithelial tumor.

Variables	DFS				OS			
	*p*	*HR*	*95%CI*		*p*	*HR*	*95%CI*	
			Lower-bound	Upper-bound			Lower-bound	Upper-bound
Masaoka-Koga stage	0.012	1.777	1.134	2.785	–	–	–	–
Histological type (4th edition)	0.025	1.758	1.075	2.874	–	–	–	–
CD20	0.003	4.74	1.673	13.434	0.014	5.086	1.391	18.594
CD204	0.004	0.154	0.043	0.547	0.002	0.169	0.056	0.607

## Discussion

This study investigated the correlation between the expression of biomarkers in the tumor microenvironment (TME) and the prognosis of thymic epithelial tumors (TETs) to explore the role of the TME in influencing TET prognosis. Research has indicated that in gastric cancer, the presence of CD20+ B cells is an independent predictor significantly associated with better patient outcomes (20). Similar positive effects have been confirmed in other malignancies such as breast cancer (21) and lung cancer (22). However, another study suggested no significant association between CD20+ cell density and survival rates in gastric and esophageal cancers (23). Furthermore, some research has shown that high infiltration of CD20+ B cells is associated with poor prognosis in renal cancer patients (24), indicating that B cells may play different roles in various types of tumors. Our results demonstrate that CD20 is an independent prognostic risk factor for both DFS and OS in TETs, with higher CD20 expression correlating with better TET prognosis.

CD204, an immune receptor expressed on M2 macrophages, has been linked to adverse outcomes and distant metastasis, including in lung cancer (25), esophageal cancer (26), and prostate cancer (27). Recent studies have proven that CD204 is a poor prognostic factor in resectable thymic carcinoma (28). This study confirms these findings and establishes CD204 as an independent prognostic risk factor for DFS and OS in TETs, with higher densities correlating with greater malignancy and poorer prognosis. This provides a target reference for future specific targeted therapies, suggesting that targeting M2 macrophages in highly malignant TETs may offer significant therapeutic benefits. These findings highlight the complex role of the TME in TET prognosis and identify potential targets for the development of novel therapeutic strategies, emphasizing the need for further research to elucidate the clinical implications of these biomarkers in TET management.

The overexpression of CD206 has been associated with poor prognosis in locally advanced breast cancer (29), and in oral squamous cell carcinoma, it promotes tumor progression by releasing EGF and inhibits antitumor immune responses (30).Additionally, it is significantly correlated with tumor recurrence in mid-to-late stage colorectal cancer (31). Results in this study indicate that the expression of CD206 is significantly negatively correlated with DFS in TETs, but not with OS. A similar situation is observed with CD47, whose overexpression has been found in various tumors and has been proven to be an independent poor prognostic factor in malignant tumors such as lung cance^r^ (32) and gastric cancer (33).However, in our study, neither CD206 nor CD47 was independent prognostic factors for TETs, showing significant correlation only with DFS. Another marker, CD8, has been shown in previous retrospective studies to have significant prognostic implications for thymic carcinoma patients due to the density of CD8+ stromal infiltrating lymphocytes (28). However, our study found no correlation between CD8 expression and DFS or OS in TETs. Results in this study may be related to the clinical characteristics of TETs, necessitating further subgroup analysis. Results from more aggressive thymic carcinomas may be more representative.

Current research posits that the WHO histological classification and Masaoka staging system play a significant role in the prognosis of thymic tumors. The majority of investigators consider the Masaoka clinical staging and WHO histological typing to be independent prognostic factors for Thymic Epithelial Tumors (TET) patients (34, 35). However, some scholars argue that the Masaoka staging and WHO classification have a number of limitations, and therefore are not independent risk factors despite their significant association with the prognosis of TET patients (36). The results of the COX regression multivariate analysis in this study indicate that Masaoka staging and WHO pathological histological typing are independent prognostic factors for Disease-Free Survival (DFS) in thymic epithelial tumors, and they are significantly correlated with Overall Survival (OS), but not independent prognostic factors for OS. A retrospective study analyzed 329 cases of thymic carcinoma and found that postoperative radiotherapy was an independent prognostic factor for thymic carcinoma (37). The single-factor analysis results of this study show that whether patients undergo postoperative radiotherapy is related to the prognosis of DFS, but not to OS. The findings differ from the former but are similar to a study conducted in Japan (38).The log-rank test results of this study reveal that patient gender, the presence of myasthenia gravis, and the administration of postoperative chemotherapy are not correlated with DFS or OS. These results are consistent with previous studies (39, 40).

This study presents an opportunity to uncover more potent cancer treatment strategies, aiming to provide a reference for understanding the characteristics of Thymic Epithelial Tumors (TETs) and assisting in clinical diagnosis and treatment. It is posited that the detection of biomarkers for TETs could serve as a valuable adjunct to Masaoka staging and WHO classification in predicting the efficacy and prognosis of TETs. This study has several limitations. Due to the rarity of TETs, the number of samples collected is relatively small. Additionally, the analysis relies on a single cohort, which limits the robustness of the findings. Including additional independent cohorts or validation datasets is essential to strengthen the conclusions. Furthermore, the markers investigated—CD20 (B cells), CD204 (macrophages), and other T-cell-related indicators—are primarily immune-related. However, it remains unclear whether potential interactions among these immune cell populations contribute to the observed outcomes.

## Conclusion

When assessing the prognosis of thymic epithelial tumors, it is essential to consider a comprehensive approach that includes Masaoka staging, WHO pathological histological typing, and the expression levels of CD20 and CD204. Clinically, immunotherapies targeting M2 macrophages may prove effective for thymic epithelial tumors with poor prognoses.

## Data Availability

The original contributions presented in the study are included in the article/**Supplementary Material**. Further inquiries can be directed to the corresponding author.
