# Supplementary material for: CD20+ and CD204+ exhibit distinct prognostic associations in thymic epithelial tumors
Source: Front Oncol. 2025 Nov 18;15:1710544. doi: 10.3389/fonc.2025.1710544 (PMC12668968; doi:10.3389/fonc.2025.1710544)
Supplement: Supplementary file 1 [file DataSheet1.docx]

**Appendix:**

D1
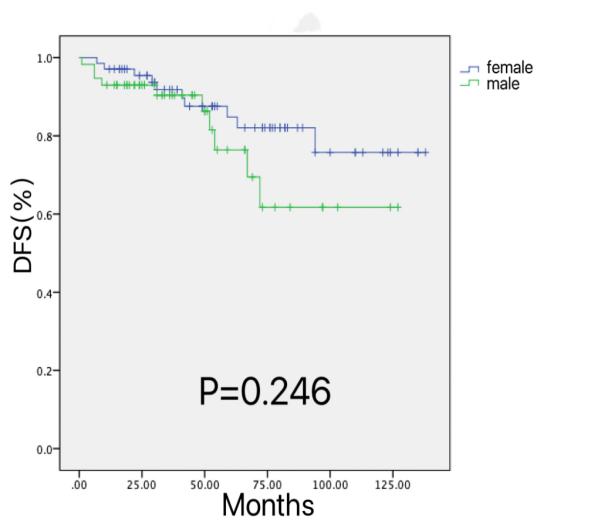
D2
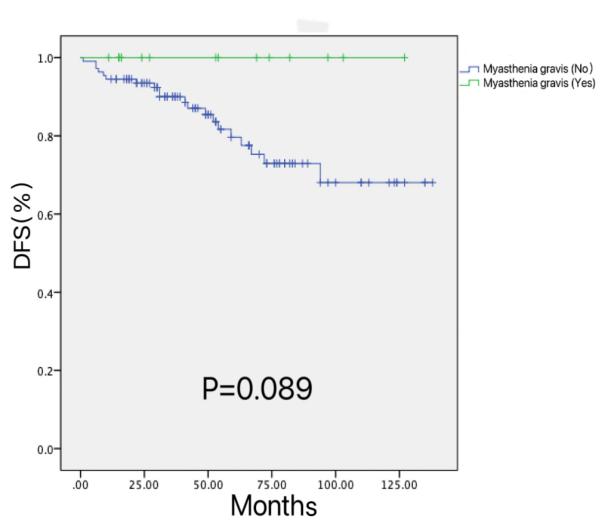


D3
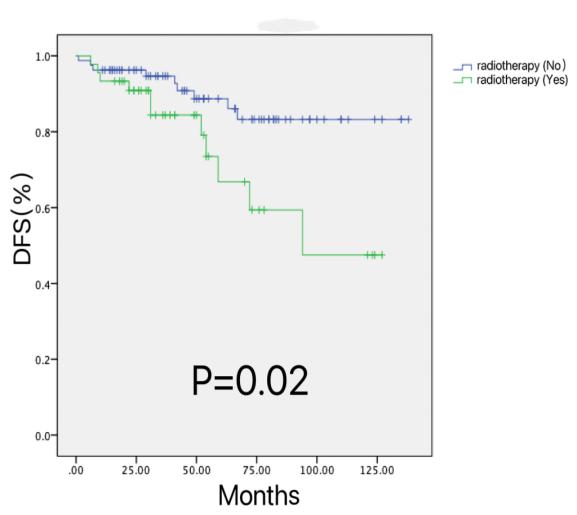
D4
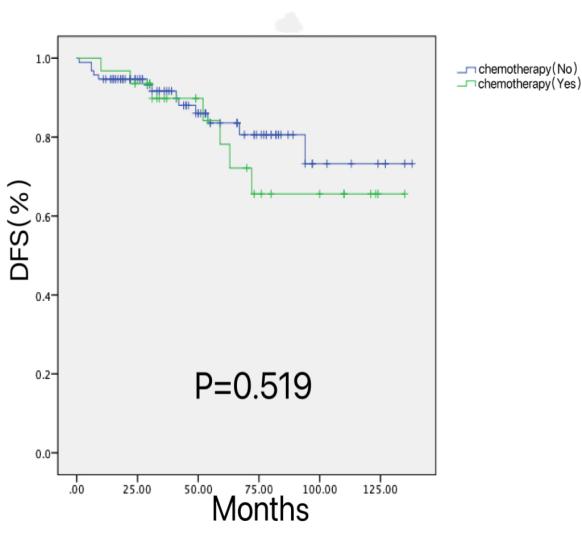


D5
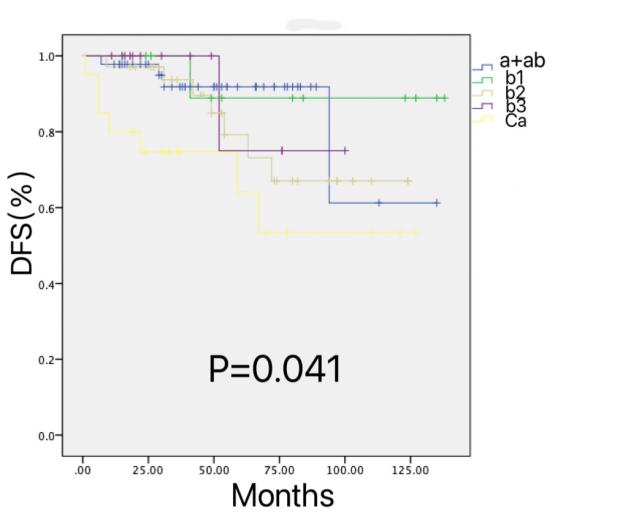
D6
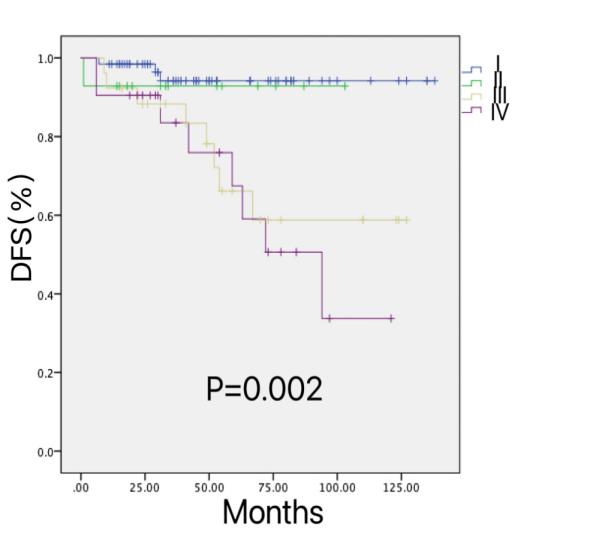


D7
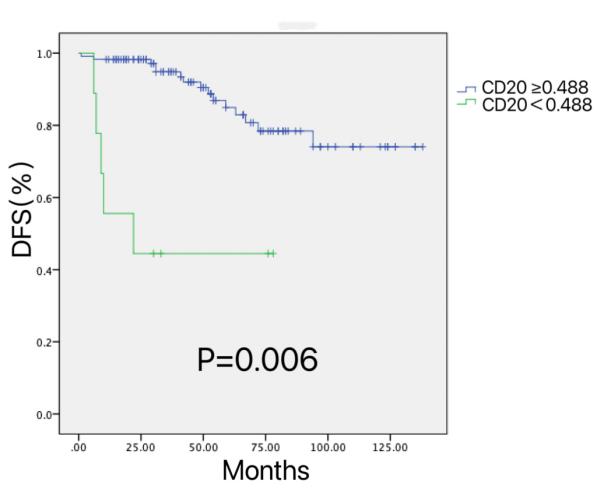
D8
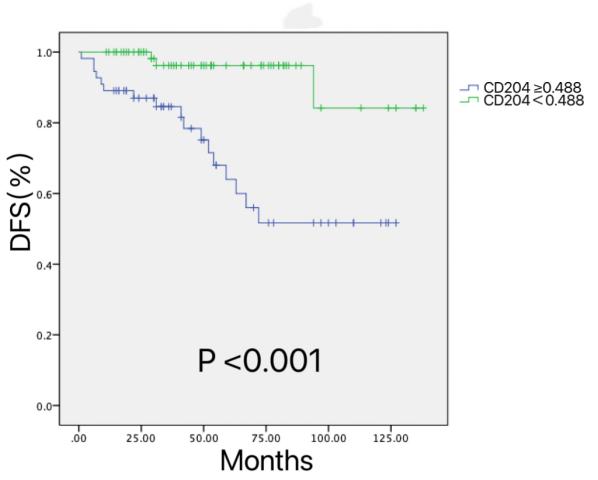


D9
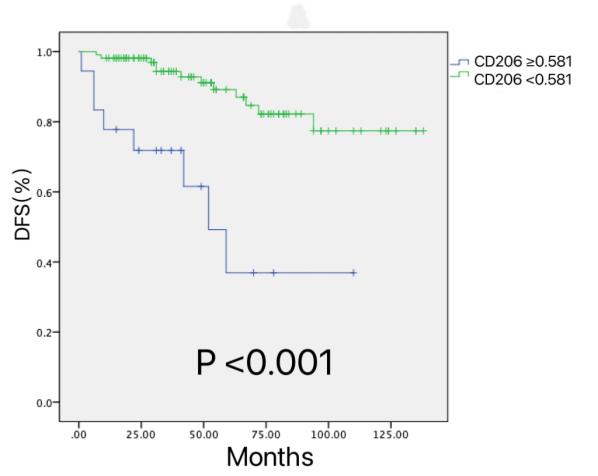
D10
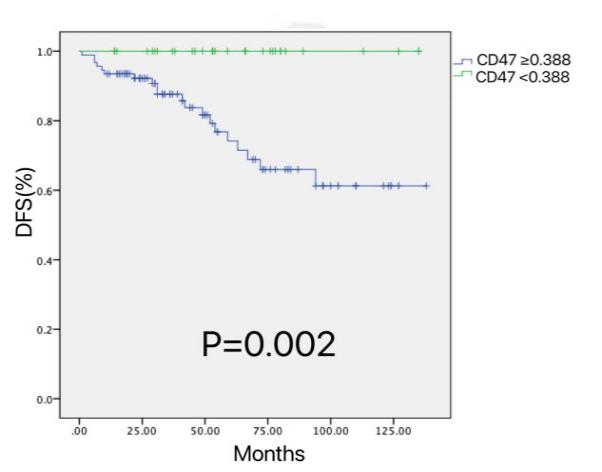


Supplementary figure group 1. survival curves for univariate analysis of different variables and DFS

（D1: Sex and DFS；D2: Myasthenia gravis (Yes) and DFS；D3: Postoperative radiotherapy and DFS；D4: Postoperative chemotherapy (Yes) and DFS；D5: Masaoka-Koga stage and DFS；D6: Histological type (4th edition) and DFS；D7: CD20 and DFS；D8:CD204 and DFS；D9:CD206 and DFS；D10: CD47 and DFS）

E1
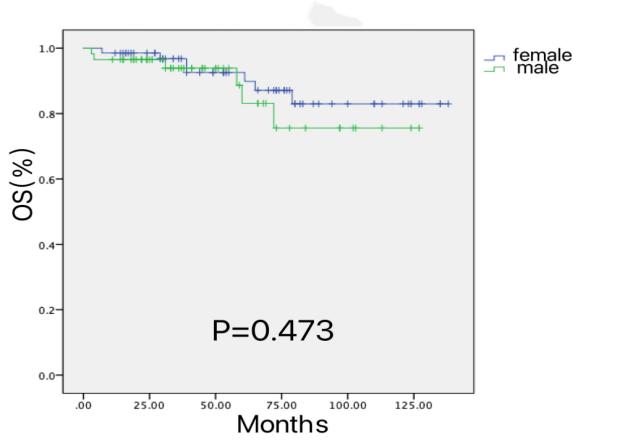
E2
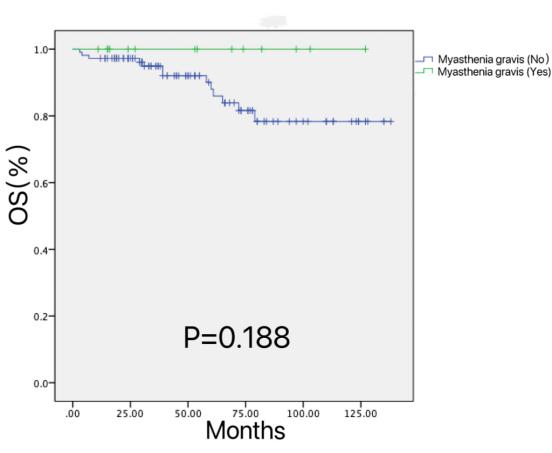


E3
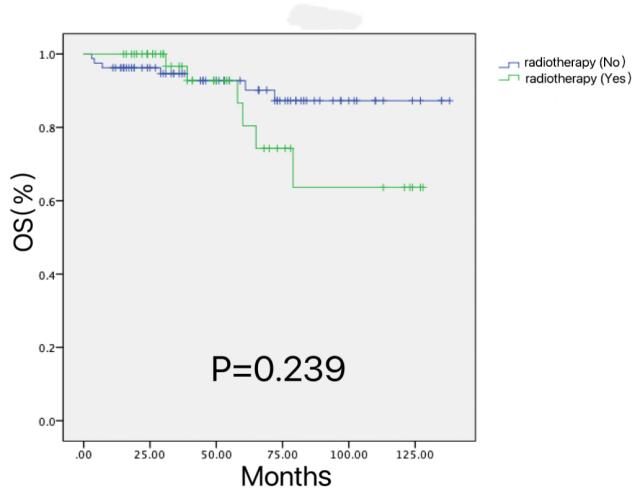
E4
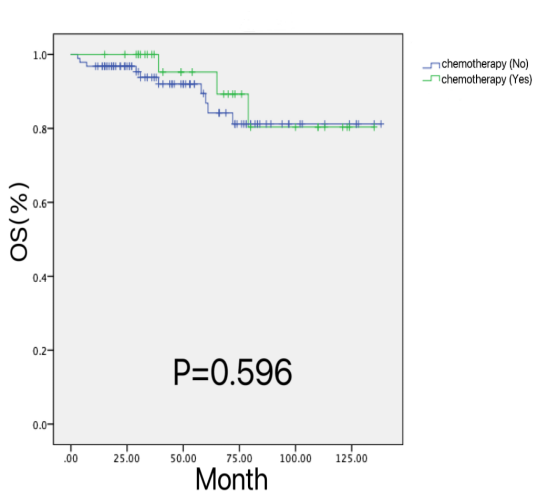


E5
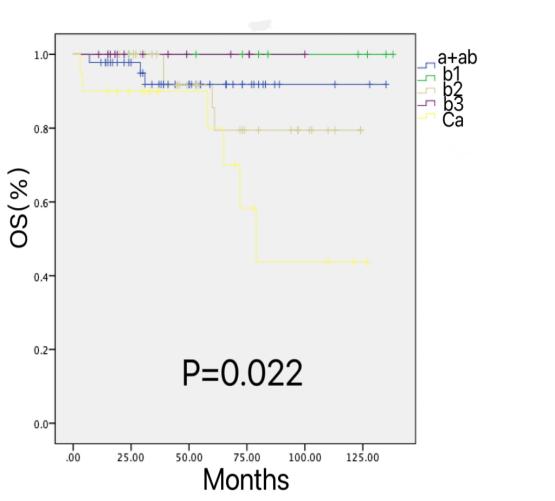
E6
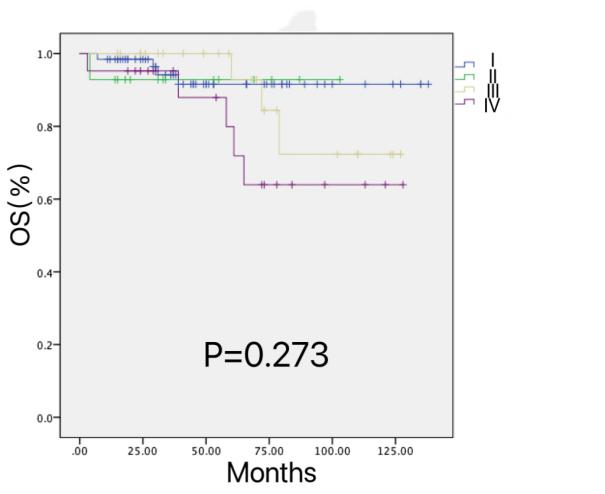


E7
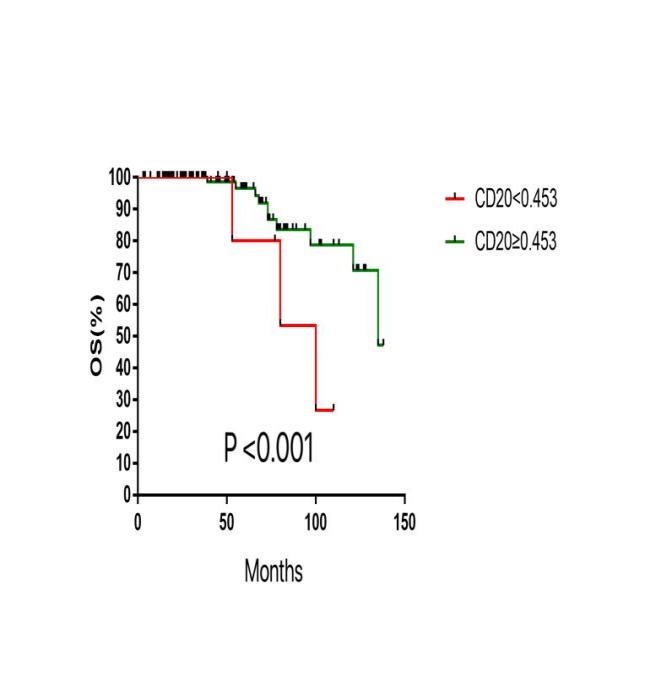
E8
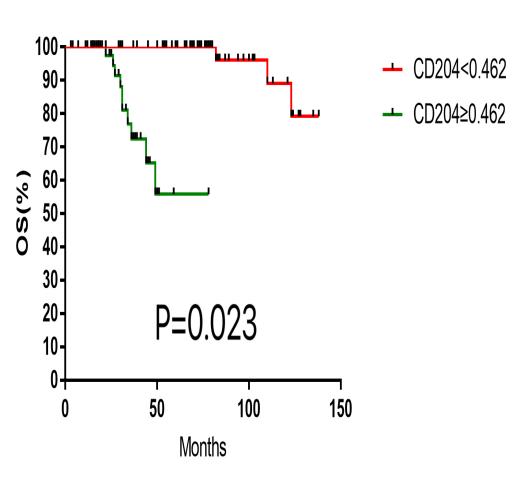


Supplementary figure group 2. survival curves for univariate analysis of different variables and DFS

（E1: Sex and OS；E2: Myasthenia gravis (Yes) and OS；E3: Postoperative radiotherapy and OS；E4: Postoperative chemotherapy (Yes) and OS；E5: Masaoka-Koga stage and OS；E6：Histological type (4th edition) and OS；E7: CD20 and OS；E8:CD204 and OS)
